# Development of an Implantable Capacitive Pressure Sensor for Biomedical Applications

**DOI:** 10.3390/mi14050975

**Published:** 2023-04-29

**Authors:** Ji-Hyoung Roh, Kyu-Sik Shin, Tae-Ha Song, Jihong Kim, Dae-Sung Lee

**Affiliations:** 1 Medical Device Development Center, Daegu-Gyeongbuk Medical Innovation Foundation, Daegu 41061, Republic of Korea; 2 Smart Sensor Research Center, Korea Electronics Technology Institute, Seongnam 13509, Republic of Korea; 3 Department of Electrical Engineering, Yeungnam University, Gyeongsan 38541, Republic of Korea

**Keywords:** capacitive pressure sensor, microelectromechanical system, biomedical applications

## Abstract

In this study, a subminiature implantable capacitive pressure sensor is proposed for biomedical applications. The proposed pressure sensor comprises an array of elastic silicon nitride (SiN) diaphragms formed by the application of a polysilicon (p-Si) sacrificial layer. In addition, using the p-Si layer, a resistive temperature sensor is also integrated into one device without additional fabrication steps or extra cost, thus enabling the device to measure pressure and temperature simultaneously. The sensor with a size of 0.5 × 1.2 mm was fabricated using microelectromechanical systems (MEMS) technology and was packaged in needle-shaped metal housing that is both insertable and biocompatible. The packaged pressure sensor immersed in a physiological saline solution exhibited excellent performance without leakage. The sensor achieved a sensitivity of approximately 1.73 pF/bar and a hysteresis of about 1.7%, respectively. Furthermore, it was confirmed that the pressure sensor operated normally for 48 h without experiencing insulation breakdown or degradation of the capacitance. The integrated resistive temperature sensor also worked properly. The response of the temperature sensor varied linearly with temperature variation. It had an acceptable temperature coefficient of resistance (TCR) of approximately 0.25%/°C.

## 1. Introduction

Pressure sensors are becoming increasingly important in a wide range of fields, such as automotive, aerospace, industrial, and robotics applications. In particular, pressure sensors are crucial in biomedical applications, including diagnostic health monitoring and precision surgery [1,2,3,4,5,6,7,8,9,10]. A good example of this case is intracranial pressure (ICP) monitoring. ICP monitoring is one of the most important assessments for managing acute neurological symptoms and has been used in the field of neurosurgery [11,12]. ICP elevation is generally caused by increased cerebrospinal fluid (CSF), intracranial blood volume, brain tumors, cerebral edema, cerebral hemorrhage, and other conditions [13,14]. In general, these conditions require immediate treatment and, in severe cases, may lead to death when left untreated. Therefore, it is crucial to accurately analyze ICP elevation [15]. The most common method for ICP monitoring involves inserting a catheter into the ventricles and connecting it to a manometer outside the body. However, this method restricts the patient’s activity and may cause physical and psychological discomfort. Moreover, there may be a possibility of significant errors during the measurement of pressure [16]. In this sense, implantable pressure sensors that can measure pressure directly should be developed for biomedical applications.

In this study, we developed a subminiature implantable capacitive pressure sensor. Pressure sensors are typically available in capacitive, piezoresistive, piezoelectric, optical, and resonant types [5]. Among them, capacitive pressure sensors have relatively high sensitivities and low temperature coefficients [17]. Furthermore, capacitive pressure sensors have low power dissipation, enabling long-term use of these sensors as insertion-type devices [18]. The proposed sensor was fabricated using microelectromechanical systems (MEMS) technology. Elastic silicon nitride (SiN) diaphragms were used for measuring capacitance change with respect to pressure, and a polysilicon (p-Si) sacrificial layer was utilized to form the diaphragms. In addition to the pressure sensor, a resistive temperature sensor was also integrated into one device to measure pressure and temperature simultaneously. The key point is that there are no additional fabrication steps or extra cost required for the integration of the temperature sensor. The resistive temperature sensor was based on the temperature coefficient of resistance (TCR) of p-Si and was fabricated simultaneously when the p-Si sacrificial layer for the pressure sensor was formed. Several reports have suggested multifunctional biosensors capable of monitoring pressure and temperature [19,20]. However, to the best of our knowledge, few studies have reported on implantable capacitive pressure sensors integrated with temperature sensors for biomedical applications. The fabricated sensor was packaged in needle-shaped metal housing with an analog-to-digital converter (ADC), making it insertable and biocompatible. The performance of the packaged sensor was characterized after its immersion in a physiological saline solution, which served as an environment similar to biological fluids.

## 2. Experimental Details

### 2.1. Design and Fabrication

**Figure 1:**
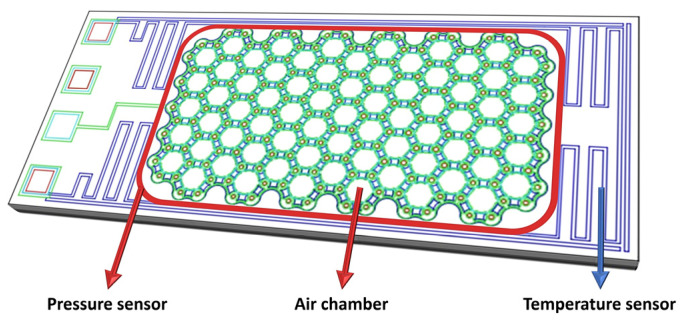
Schematic of the proposed device comprising pressure and temperature sensors.

The proposed capacitive pressure sensor utilizes elastic SiN diaphragms with supporting edges and a fixed SiN bottom layer that can form sealed air-filled chambers. When pressure is applied, the deformation of the diaphragms occurs, which results in a change in capacitance. The air chambers are connected to each other in parallel for effective measurement of pressure with a combined capacitance. The supporting edges for the diaphragms were designed to have honeycomb-shaped structures because the array-type honeycomb structure, an arrangement of hexagons, has the advantage of high durability [21]. In order to form the air chamber, a p-Si sacrificial layer is utilized in the fabrication process. The SiN diaphragm and bottom layer are deposited on and below the patterned p-Si sacrificial layer, and the sacrificial layer is removed through xenon difluoride (XeF_2_) isotopic etching. When the p-Si is patterned, the resistive temperature sensor is also formed simultaneously. P-Si enables the TCR to be regulated to negative, zero, and positive values by varying the doping concentration, thus making it suitable for fabricating both the sacrificial layer and the resistive temperature sensor [22]. A meander-shaped p-Si resistor pattern was designed for the temperature sensor with a resistance of 5 kΩ. It surrounds the pressure sensor along the edge of the device. A schematic of the proposed sensor is shown in Figure 1. The size of the unit device comprising the pressure and temperature sensors is 0.5 × 1.2 mm.

**Figure 2:**
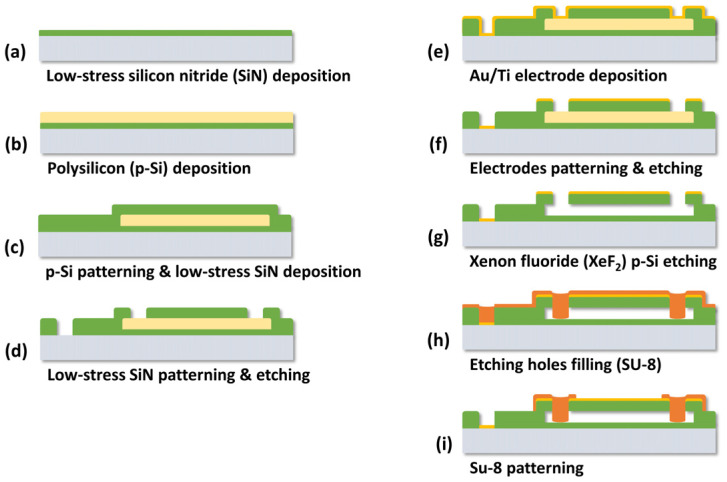
Fabrication process of the proposed sensor.

The sensor was fabricated using MEMS technology. Figure 2 shows the manufacturing process of the proposed sensor. A low-resistance Si wafer was used as a substrate with a resistance from 0.001 to 0.005 Ω. The Si substrate itself served as a bottom electrode, eliminating the need for additional fabrication steps. A low-stress SiN film was deposited on the silicon substrate, followed by the formation of a p-Si film through low-pressure chemical vapor deposition (LPCVD). The p-Si film acted as the sacrificial layer in the pressure sensor and the resistor in the temperature sensor. Therefore, a boron (B)-doping process was carried out to control the sheet resistance of the p-Si film. The sheet resistance of the p-Si measured using a four-point probe was 7.8 Ω/square. The patterning of the p-Si for the sacrificial layer and the resistor was achieved simultaneously. After that, an additional low-stress SiN film was deposited to form the diaphragms and the supporting edges of the air chambers. The SiN film was partially etched by photolithography and reactive ion etching (RIE) to form contact holes to the Si substrate for the bottom electrode and etching holes for removing the sacrificial layer. Thereafter, the top and bottom metal electrodes were formed by sputter deposition of a thin film of gold/titanium (Au/Ti) and photolithography. The p-Si sacrificial layer was removed using XeF₂ isotropic etching through the etching holes to construct the diaphragm of the pressure sensor. Lastly, the etching holes were then filled with SU-8 photoresist.

### 2.2. Packaging

**Figure 3:**
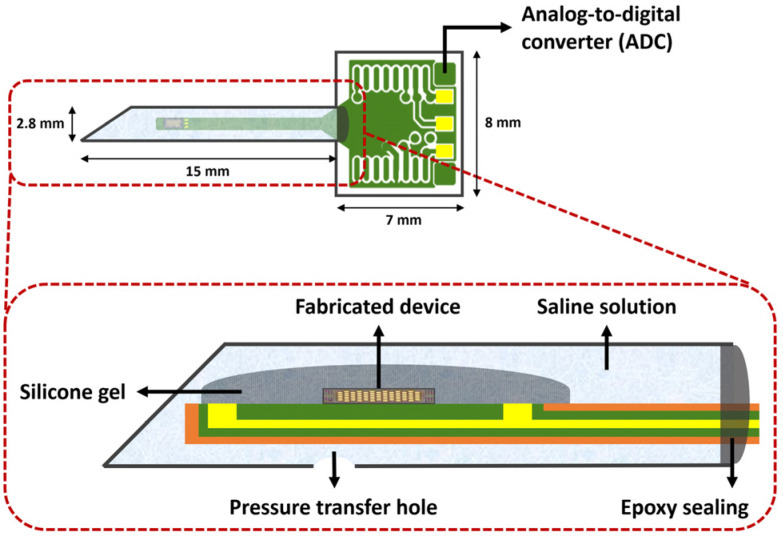
Schematic of the packaged sensor.

In this study, the proposed sensor was developed for implantable biomedical applications such as ICP monitoring. Therefore, the packaging of the sensor was designed to be insertable and biocompatible. Figure 3 shows the schematic of the packaged sensor. The fabricated sensor was attached by silicone epoxy to a suitably designed printed circuit board (PCB) with a long, narrow shape for insertion. The ADC chip (AD7745, Analog Devices, Wilmington, MA 01887, USA) was also integrated on the other end to transform the analog signal output from the sensor to its digital equivalent. After the wire-bonding process for electrical connections, the bonding wires were also covered with epoxy resin. The part of the PCB where the fabricated sensor was placed was packaged in needle-shaped metal housing with a diameter of about 2.8 mm; this is suitable for inserting into biological organs such as the brain. In order to prevent transplant rejection, a Ti thin film was sputtered over the metal housing. A hole was drilled on the surface to enable pressure transmission into the housing, and physiological saline was injected through the hole. The physiological saline was used as a biocompatible material that transfers the pressure to the sensor in biological fluids, such as CSF, when inserted into organs. However, because physiological saline is conductive, the sensor had to be insulated without interrupting the pressure transfer. Therefore, the sensor was coated with silicone gel for insulation before the metal housing packaging. The silicone gel facilitated stable pressure transfer to the sensor by changing its shape according to the applied pressure. 

## 3. Results and Discussion

### 3.1. Imaging

**Figure 4:**
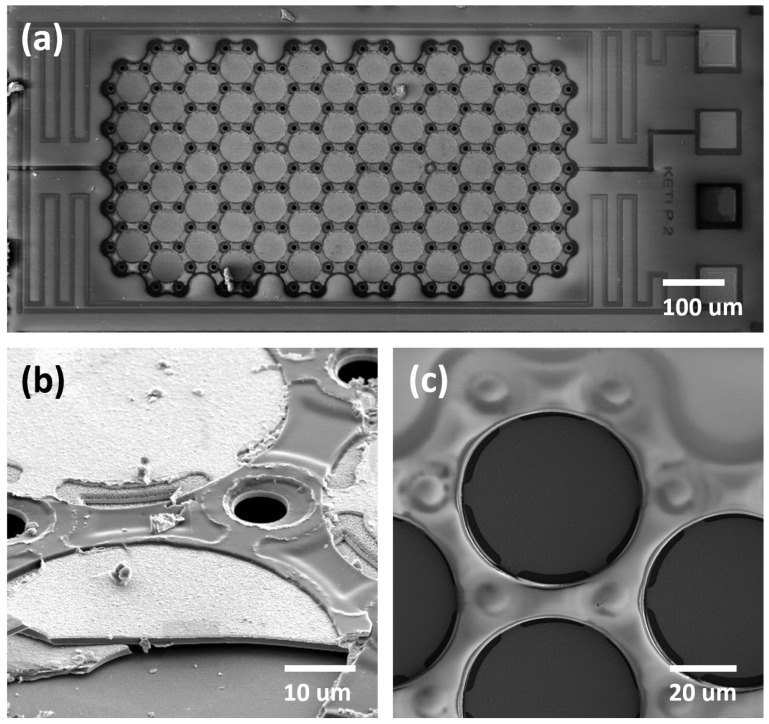
SEM image of (**a**) the fabricated sensor before the filling of the etching holes, (**b**) the air chamber after bursting, and (**c**) the diaphragms after the filling.

The fabricated sensor was examined using scanning electron microscopy (SEM). Figure 4a shows the top-view SEM image of the fabricated sensor before the etching holes were filled. The formation of the array-type SiN diaphragms for the capacitive pressure sensor can be seen clearly. In addition, the meander-shaped p-Si resistive temperature sensor pattern integrated around the pressure sensor also appears. The diaphragms after the filling are also presented in Figure 4c. The formation of the air chamber was verified through SEM after one of the chambers was broken; the results (Figure 4b) confirmed the successful formation of the air chamber through XeF_2_ isotopic etching. 

**Figure 5:**
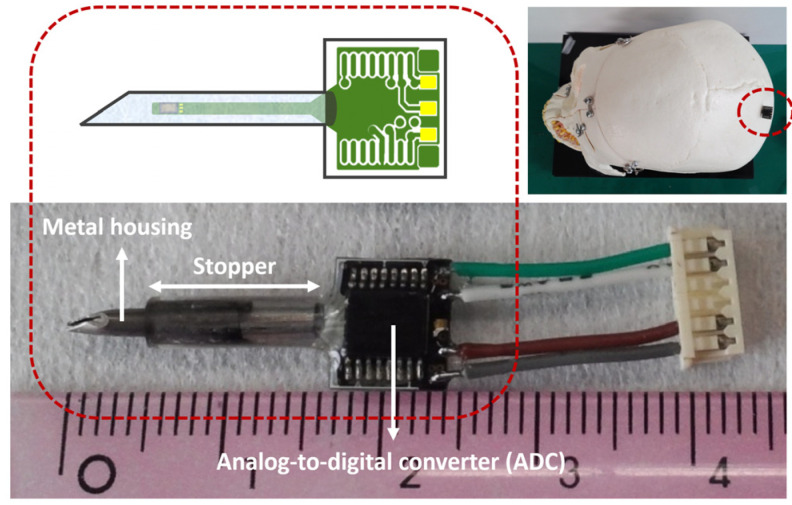
Image of the developed sensor after packaging.

Figure 5 depicts the image of the developed sensor after packaging. The long and narrow-shaped PCB with the fabricated sensor was successfully packaged in the needle-shaped metal housing. As mentioned earlier, the ADC was attached to the other end of the PCB. A rubber ring stopper shown in Figure 5 was additionally prepared. If needed, it can be utilized to ensure that the metal needle is accurately positioned as desired (e.g., at the cerebral ventricle of the subject). The top right inset of Figure 5 shows the conceptual image of the packaged sensor if applied for ICP measurements. 

### 3.2. Characterizations

#### 3.2.1. Output Characteristics before Packaging

**Figure 6:**
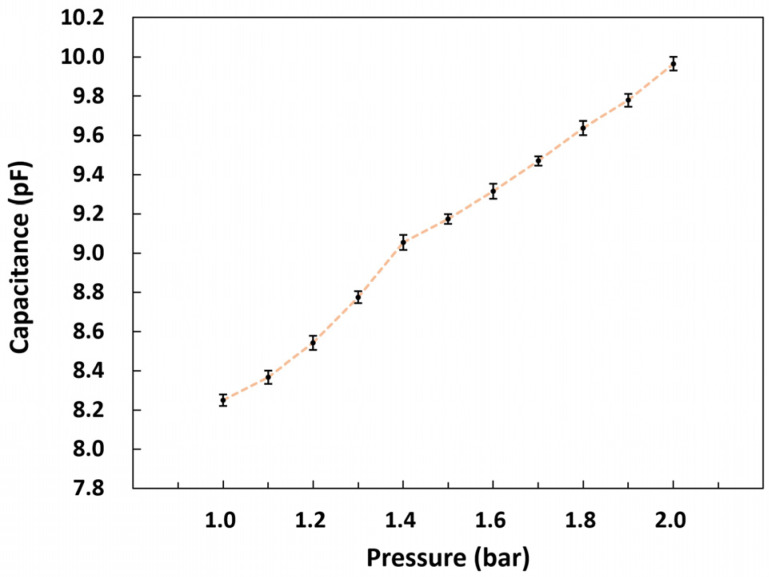
Output characteristics of the pressure sensors before packaging. The points represent the average values for 10 randomly chosen sensors. The error bars indicate standard deviation.

Before packaging, 10 devices were randomly chosen and characterized using an LCR meter and a pressure chamber to evaluate the device-to-device reproducibility. The pressure chamber had ports for electrical connection to the outside and for applying pressure. The pressure in the chamber was controlled precisely with a commercial digital pressure controller (Ruska series 7000, Fluke Calibration, Everett, WA 98203, USA) and varied within the range of 1–2 bar. Figure 6 shows the change in the capacitance of the fabricated pressure sensors as a function of pressure. The points in the figure represent the average values for 10 devices, and the error bars denote standard deviation. It can be confirmed that 10 randomly chosen devices exhibited quite a similar device-to-device performance with small variations.

#### 3.2.2. Leakage Current

**Figure 7:**
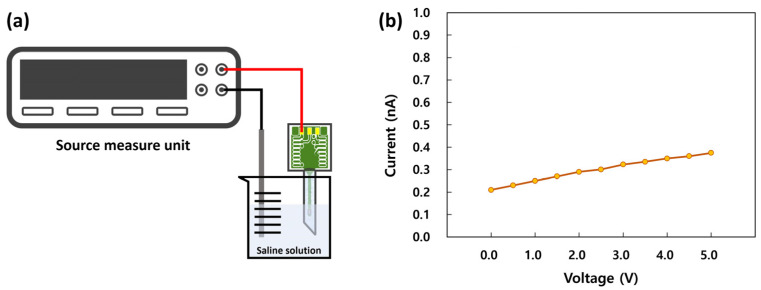
(**a**) Schematic of the leakage current measurement set-up. (**b**) Leakage current characteristic of the packaged sensor.

Because the proposed sensor was packaged with the intention of use in environments with biological fluids, the metal housing was filled with physiological saline to ensure biocompatibility and facilitate pressure transfer. Due to this, as discussed earlier, the sensor was coated with silicone gel for insulation before packaging. A leakage current test was performed to assess the insulation characteristics prior to the performance characterization of the packaged sensor. As shown in Figure 7a, the packaged sensor was immersed in a physiological saline solution to emulate environments with biological fluids, and the leakage currents between the Ag/AgCl and sensor electrodes were measured using a source measure unit (SMU). Voltages ranging from 0 to 5 V were applied through the SMU, and the resulting leakage currents ranged from 0.21 to 0.37 nA, as shown in Figure 7b. These results indicated that the packaged sensor exhibited excellent insulation performance, and there would be no leakage problem in the performance characterization procedure.

#### 3.2.3. Performance of the Pressure Sensor

**Figure 8:**
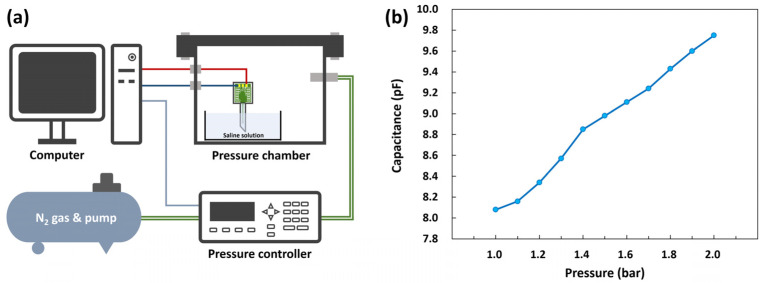
(**a**) Schematic of the pressure measurement system. (**b**) Performance of the pressure sensor.

A pressure measurement system was constructed to measure the output capacitance of the packaged sensor. Figure 8a shows the schematic of the pressure measurement system. The packaged sensor was immersed in a physiological saline solution in a petri dish placed in the pressure chamber. The pressure in the chamber was increased from 1 bar to 2 bar, and the changes in the output capacitance of the sensor as a function of pressure were recorded using a computer connected through an evaluation board. 

Figure 8b illustrates the change in capacitance with respect to pressure. The capacitance of the sensor increased as the pressure in the chamber increased. The sensitivity of the sensor was calculated to be approximately 1.73 pF/bar, which is sufficiently high for detecting pressure variations. One thing to note is that the inflection point appeared around 1.4 bar in Figure 8b. This could be caused by the contact of the low-stress SiN diaphragm to the bottom. When low pressure is applied to a capacitive pressure sensor, the diaphragm of the sensor simply deforms without contact (non-contact mode). However, when the pressure reaches a certain level, the diaphragm contacts the bottom (contact mode). In the non-contact mode, the capacitive sensor exhibits a non-linear response that is inversely proportional to the distance between the top and bottom. On the other hand, in contact-mode, a linear response can be achieved because the capacitance increases with the gradual increase in the contact area between the diaphragm and the bottom [23,24,25,26]. In this study, the fabricated sensor exhibited a non-linear response for pressures up to 1.4 bar; beyond this the response was linear. Therefore, the diaphragm transition from non-contact to contact mode is considered to occur at approximately 1.4 bar.

**Figure 9:**
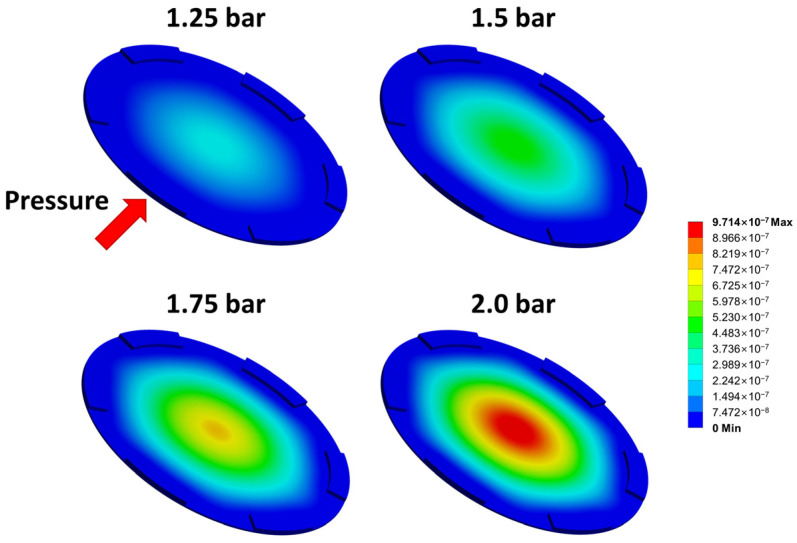
Finite element analysis for the diaphragm at various absolute pressures (1.25, 1.5, 1.75, and 2.0 bar).

The finite element analysis (FEA) using ANSYS software was carried out to validate the deformation of the pressure sensor’s diaphragm. The three-dimensional model of the diaphragm was created, and the absolute pressures ranging from 1.0 to 2.0 bar were applied to the top surface of the diaphragm. The FEA results of the diaphragms at various pressures (1.25, 1.5, 1.75, and 2.0 bar) are shown in Figure 9; here, the bottom structures are not shown, and the diaphragms are viewed from the bottom. When pressures of 1.5 bar and 1.75 bar were applied to the surface of the diaphragm, the deformations were determined to be about 500 nm and 750 nm, respectively. Considering the actual distance between the diaphragm and the bottom of the pressure sensor, which is estimated to be from 600 to 700 nm, the contact is supposed to occur at between 1.5 and 1.75 bar. The inflection point from the actual measurements in Figure 8b appeared around 1.4 bar; this is slightly lower than the FEA result. This deviation is thought to be attributed to the structural difference in the fabricated pressure sensor from the ideal design, which can be caused by the XeF_2_ isotopic etching.

#### 3.2.4. Hysteresis of the Pressure Sensor

**Figure 10:**
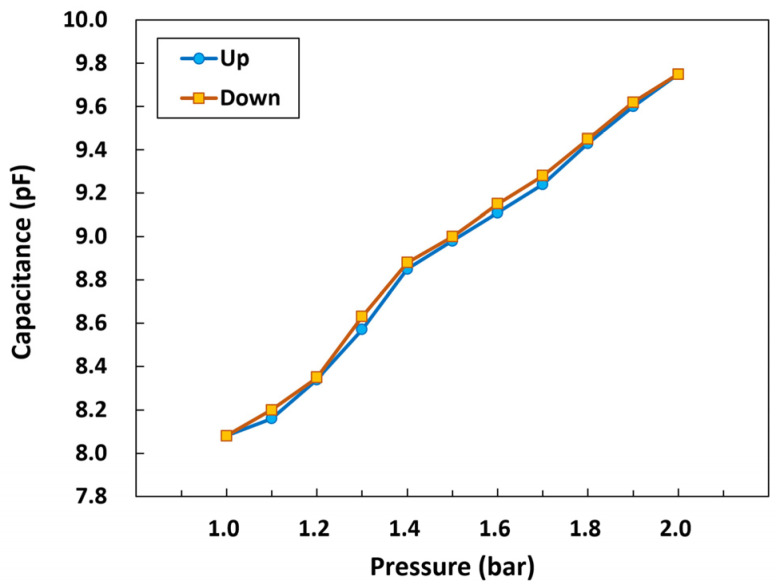
Hysteresis characteristic of the pressure sensor.

The hysteresis characteristic of the packaged pressure sensor was also evaluated. In the evaluation process, the pressure in the chamber was varied within the range of 1–2 bar, and the capacitance of the packaged sensor immersed in physiological saline was measured repeatedly by increasing and decreasing the pressure. As shown in Figure 10, the results indicated that the developed pressure sensor exhibited a reversible response with a small hysteresis. The calculated maximum absolute value of the hysteresis was about 1.7% at approximately 1.3 bar. 

#### 3.2.5. Long-Term Stability of the Pressure Sensor

**Figure 11:**
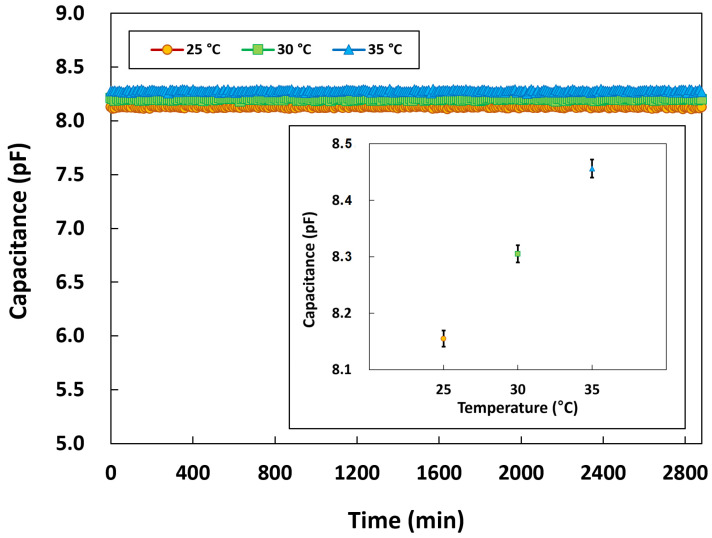
Long-term stability characteristics of the pressure sensor at different temperatures.

Long-term stability tests were conducted for 48 h to ensure that the packaged pressure sensor can be used continuously in biomedical applications. Although it is known that capacitive pressure sensors ideally tend to exhibit low temperature dependence, the tests were carried out under different temperature conditions to clarify the practical stability of the developed sensor. As in the aforementioned experiments, the tests involved immersing the packaged sensor in a physiological saline solution, and the pressure chamber was placed in a temperature chamber to maintain the temperature conditions. The temperatures were set at 25, 30, and 35 °C, and the capacitance of the sensor was measured every 10 min. Figure 11 shows the long-term stability characteristics of the packaged pressure sensor at 25, 30, and 35 °C. The pressure sensor exhibited excellent long-term stability, with normal operation sustained throughout the tests without any insulation breakdown or capacitance degradation at all temperatures. The inset of Figure 11 shows the average capacitance values of the pressure sensor for 48 h with the error bars indicating standard deviation. The sensor’s capacitances remained highly stable, with a variation of less than ±0.28%. As expected, the pressure sensor exhibited low temperature dependence. The capacitance of the pressure sensor increased by about 0.015 pF when the temperature increased by 1 °C. These results indicated that the proposed sensor could operate normally during in vivo experiments.

#### 3.2.6. Performance of the Temperature Sensor

**Figure 12:**
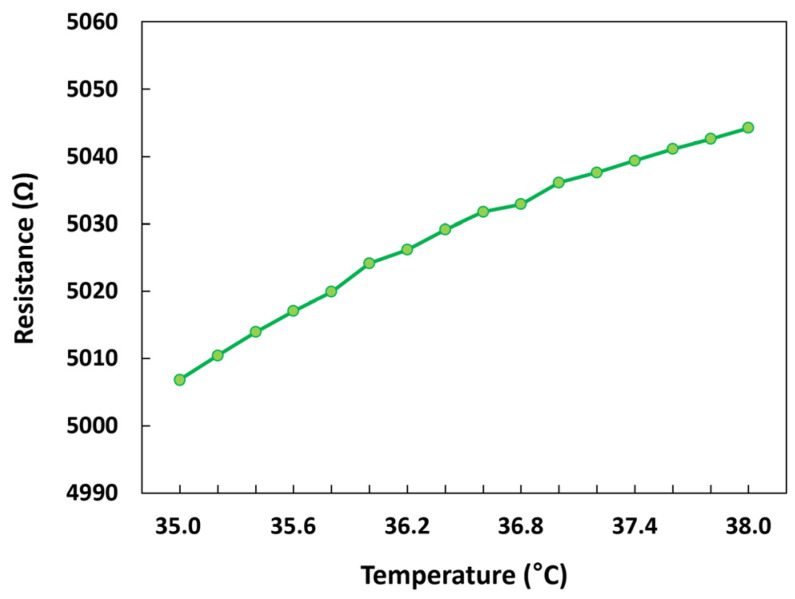
Performance of the temperature sensor.

The resistive temperature sensor integrated with the pressure sensor was also characterized in the temperature chamber. The temperature in the chamber was varied within the range of 35–38 °C, which is similar to human body temperature. Figure 12 illustrates the change in resistance with respect to temperature. The actual resistance of the sensor was approximately 5 kΩ, which was consistent with the initial design. The resistance increased linearly as the temperature in the chamber increased. *TCR*, a key parameter for a temperature sensor, can be used to represent the sensitivity and obtained from the slope of the resistance–temperature curve. *TCR* is formally defined as follows:

(1) $$TCR=\frac{1}{R}\frac{dR}{dT}.$$

The *TCR* of the temperature sensor was calculated to be approximatelyt 0.25%/°C, which is slightly lower than that of the commercial platinum temperature sensors (0.39%/°C) [27,28]. However, it should be noted that the developed temperature sensor was integrated without requiring additional fabrication steps, and the p-Si deposited for the sacrificial layer of the pressure sensor also served as the temperature-sensing material in the device. Considering this advantage, the achieved TCR value of the developed sensor is acceptable for temperature sensing. 

**Figure 13:**
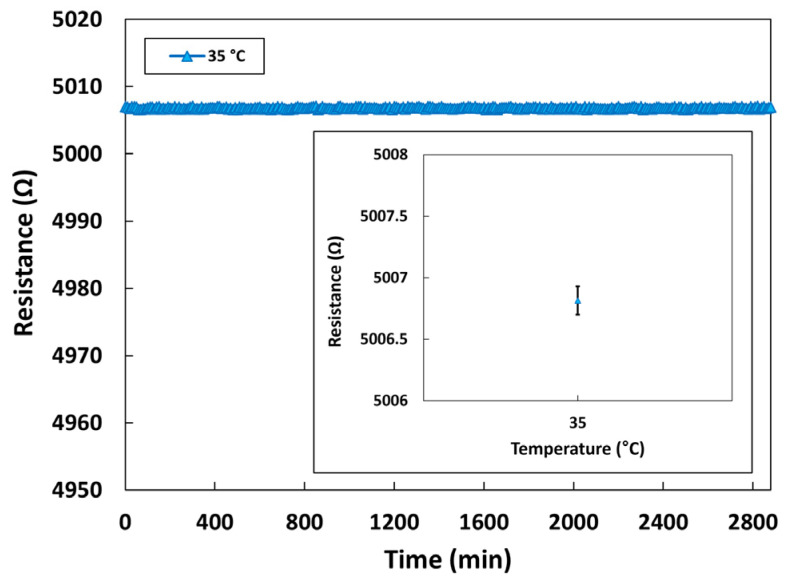
Long-term stability characteristics of the temperature sensor.

Long-term stability test for the temperature sensor was also performed for 48 h at 35 °C, and the result is shown in Figure 13. As expected, the temperature sensor exhibited normal operation without the insulation breakdown or resistance degradation. The average resistance value of the temperature sensor for 48 h is presented in the inset of Figure 13. The error bars represent the standard deviation. The temperature sensor operated stably and exhibited very small fluctuations throughout the measurements.

## 4. Conclusions

In this study, we developed an implantable capacitive pressure sensor based on MEMS technology for biomedical applications such as ICP monitoring. In addition, we successfully integrated a resistive temperature sensor with the pressure sensor without additional fabrication steps or extra cost. The developed sensor mounted on the suitably designed PCB with the ADC chip was enclosed in biocompatible, needle-shaped metal housing to facilitate insertion during medical procedures. After immersion in a physiological saline solution, the packaged sensor was characterized, emulating a biological fluid environment. The packaged sensor with silicone gel exhibited excellent insulation characteristics. The packaged pressure sensor also exhibited great performance with a sensitivity of approximately 1.73 pF/bar and a hysteresis of approximately 1.7%. Furthermore, the pressure sensor operated normally for 48 h without insulation breakdown or capacitance degradation. The integrated temperature sensor responded satisfactorily to temperature variations, with an acceptable TCR of approximately 0.25%/°C. This study may serve as a foundation for further studies involving in vivo experiments: for example, measuring ICP in rats. Further miniaturization of the packaging by optimizing the metal housing and the PCB will be left for future work. The integration with wireless data transmission capabilities will also be essential. Further studies and research on the development of wireless communication modules, power modules, and interfaces between the sensor and a wireless communication module will be needed to realize the wireless system.

## Data Availability

Not applicable.
